# Can Science Resolve the Doubt?

**Published:** 1890-11-08

**Authors:** 


### The Doctor's Armchair.

### CAN SCIENCE RESOLVE THE DOUBT?

SOME of the more adventurous spirits among magazine and newspaper writers have lately been pushing forward a question of the deepest interest to all maidens and bachelors. May a woman propose? The mere suggestion almost takes the breath away! But if we begin to look for anything which can properly be called a conclusive reason why a woman should not propose, we do not find our search very quickly successful. Reasons there are by the dozen which will satisfy those men and women who live by conventional rules. But conventional rules are made for conventional minds, not for those who have courage enough for independent and original thought. Many persons hate the original thought of their own generation; but with the oddest inconsistency they will swear by, or even die for, the original thought of one or two thousand years ago. We have, therefore, this curious spectacle, that large numbers of people, because they live in the nineteenth century, pin their faith to the thought of the tenth or the first; but those same persons if they had lived in the tenth or the first century would have despised and repudiated the very thought they now hold in such veneration. We do not write with the object of convicting conventional people of inconsistency, but of insisting that if original thought be generally good to live by, the thought of honest and capable men in the nineteenth century may be as practically useful as that of the same sort of men in the tenth or the first. Nay, it is quite probable that on some questions the nineteenth century thinker may have certain advantages over his intellectual peer who lived in the days of Julius Cæsar. Without further pleading, may we not conclude that there are questions which the men and women of this generation are entitled to reconsider; and that this, of the liberty of women to choose their own husbands, and, if need be, to make the marriage proposal, is emphatically one of them?

The writer has great faith in the scientific, as opposed to the conventional, spirit. Not that he holds the average man of science to possess a better judgment in all common things than other persons of equal strength of mind. But the scientific man pursues his special work with a temper of soul of the very highest intellectual quality; and if he were habitually to employ his laboratory temper on all the social, political, and general questions of his time he would certainly arrive at juster conclusions than the ordinary man reaches. The scientific temper is not only the best for science, but for all other questions. The pity is that the average scientific man brings that temper into discredit exactly as the religious man brings religion into discredit—by keeping it for special occasions. Let us try the scientific temper, for instance, on this particular question, the mere enunciation of which has sent such a shudder through the ranks of a half petrified conventionalism! Mr. Darwin shall furnish us with a fact or two in the history of creatures very nearly related to ourselves. The loves and wooings and weddings of birds have often furnished inspiration to the poet. May we not seek from them, with equal propriety, a little light on the all-important social question we are now discussing?

"Naturalists," says Mr. Darwin, "are much divided with respect to the object of the singing of birds. Few more careful observers ever lived than Montagu, and he maintained that the males of song birds and of many others do not in general go in search of the females; but, on the contrary, their business in the spring is to perch on some conspicuous spot, breathing out their full and amorous notes, which, by instinct, the female knows, and repairs to the spot to choose her mate. Mr. Jenner Weir informs me that this is certainly the case with the nightingale. Bechstein, who kept birds during his whole life, asserts that the female canary always chooses the best singer, and that in a state of nature the female finch selects that male out of a hundred whose notes please her most. There can be no doubt that birds closely attend to each other's song. Mr. Weir has told me of the case of a bullfinch which had been taught to pipe a German waltz, and who was so good a performer that he cost ten guineas; when this bird was first introduced into a room where other birds were kept and he began to sing, all the others, consisting of about twenty linnets and canaries, ranged themselves on the nearest side of their cages, and listened with the greatest interest to the new performer." From these considerations and from other facts observed by the writer himself, there seems to be no doubt that in the case of some birds a condition of things obtains which is the exact opposite of what is seen among the human kind. With us, women are more beautiful by nature than men; and most of them do their best to add to their beauty by art. In ultimate analysis the object of this seems to be to make themselves pleasing to men in order that they may be chosen as wives. Among certain families of birds it is the male that is beautiful by nature, whilst the female is quite plain and insignificant. The male is not only naturally beautiful but has the art to play a great variety of antics in sight of the female in order to enhance his natural good looks. By way of compensation, the female is endowed with the critical faculty and the power of choice. The coy male, in spite of his good looks and power of song, must wait until he is asked before he can fly off with his mate. If, as sometimes happens, he is not asked at all, he must do the best he can in his bachelor lodgings, at least for that season.

Now, we never heard of any harm happening to the morals of the bird community as the result of this particular arrangement; and it is difficult to imagine how any unhappy consequences could possibly spring from it. What strikes us, indeed, in thinking of the case of the birds is that it matters little whether the male or the female has the primary right of proposal. The chief thing is that the proposal be made by one or the other, and that the marriage shall be happy. Speaking as a man, the writer sees no valid reason why the custom of the birds should not prevail universally among men, instead of the practice which now obtains. It would at least be more polite and more chivalrous for men to wait until they were asked before urging their love upon women. But that is, perhaps, a counsel of perfection. What seems of immediate moment is the consideration that those countries which are most highly civilized have agreed to regard woman as the equal of man. But she cannot be his equal in practice if in the most momentous of all life's circumstances she is compelled always to wait for his initiative. If woman be, indeed, man's equal, it is clear, that in reason at least, she has exactly the same right to make a proposal of marriage as he has. But this other case often happens, and must be duly considered, that a particular woman is decidedly the superior of a particular man, whom, notwithstanding, she would like to marry. There are few persons who cannot call to mind instances in which two human beings are exactly suited to one another, and each is very much in love with the other, and yet because the woman is superior to the man in intellect, wealth, or social position, they are kept apart and both rendered miserable thereby. Why, in such a case, should not a woman be countenanced by society in frankly saying to a man that she loves him, and would like to marry him? Is it because men are so base and unchivalrous that they cannot be trusted to receive such offers, and to lock them up honourably and for ever in their own breasts? That we do not believe. Women hear the confessions of men; and when they receive them unfavourably, they do not think of proclaiming upon the house-tops that they have received and refused honourable proposals from honourable suitors. They respect the confidence of those who admire them, and keep their secret right loyally. There is no doubt that the average man would do the same. The present condition of society seems to demand the general equalisation of the sexes; and if that be so, it is difficult to see, with anything like clearness, why a woman should be a man's equal in every other point but that particular one which is the most momentous of all to her life's happiness.

